# Plasmonic-enhanced photocatalysis reactions using gold nanostructured films

**DOI:** 10.1039/d0ra03858j

**Published:** 2020-06-10

**Authors:** Mohammed A. Ibrahem, Bassam G. Rasheed, Rahman I. Mahdi, Taha M. Khazal, Maryam M. Omar, Mary O'Neill

**Affiliations:** Laser Sciences and Technology Branch, Applied Sciences Department, University of Technology Baghdad Iraq mohammed.a.ibrahem@uotechnology.edu.iq; Laser and Optoelectronic Engineering Department, College of Engineering, Al-Nahrain University Baghdad Iraq; Nanotechnology and Advanced Materials Research Centre, University of Technology Baghdad Iraq; School of Science and Technology, Nottingham Trent University Clifton Lane Nottingham NG11 8NS UK

## Abstract

This work shows the enhancement of the visible photocatalytic activity of TiO_2_ NPs film using the localized surface plasmonic resonance of Au nanostructures. We adopted a simple yet effective surface treatment to tune the size distribution, and plasmonic resonance spectrum of Au nanostructured films on glass substrates, by hot plate annealing in air at low temperatures. A hybrid photocatalytic film of TiO_2_:Au is utilized to catalyse a selective photodegradation reaction of Methylene Blue in solution. Irradiation at the plasmonic resonance wavelength of the Au nanostructures provides more effective photodegradation compared to broadband artificial sunlight of significantly higher intensity. This improvement is attributed to the active contribution of the plasmonic hot electrons injected into the TiO_2_. The broadband source initiates competing photoreactions in the photocatalyst, so that carrier transfer from the catalyst surface to the solution is less efficient. The proposed hybrid photocatalyst can be integrated with a variety of device architectures and designs, which makes it highly attractive for low-cost photocatalysis applications.

## Abstract 2

This work shows the enhancement of the visible photocatalytic activity of TiO_2_ NPs film using the localized surface plasmonic resonance of Au nanostructures.

## Graphical abstract

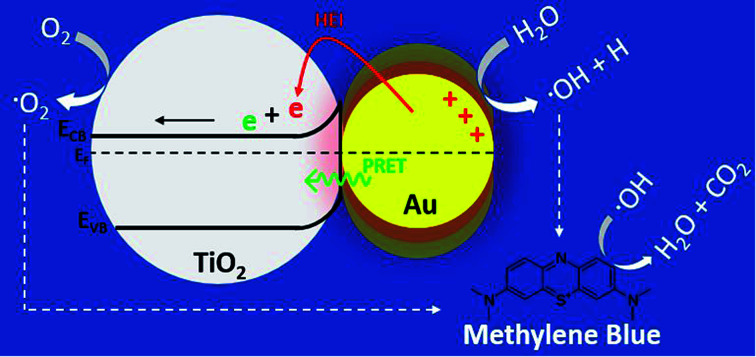

## 1 Introduction

In recent times, environmental contamination has become a serious threat not only to human living conditions but also to our planet. Therefore, great scientific and technological efforts have been made to adopt new cost-effective and environmentally friendly remediation techniques to overcome pollution issues. An example is harvesting the sunlight as an energy source to photocatalyse organic contaminants in water such as endocrine-disrupting chemicals, some pharmaceutical effluents, and personal care products which are hard to process by conventional treating methods.^1,2^ However, conventional photocatalysis systems mainly use wide bandgap semiconductors, such as TiO_2_ with a band gap of 3.2 eV,^3^ as the main catalyst. This has drawbacks, such as its ability to harvest only 5% of the incident solar light reaching the earth's surface (UV band spectrum) and also its high charge carrier recombination rate. To resolve these issues, scientists have focused on improving the photocatalysis performance by re-engineering the semiconductor's bandgap so that it can respond to the visible light. Various methods have been used such as doping and/or mixing with other materials to enhance the visible photocatalytic activity.^4–6^ However, charge trapping often hampers the efficiency and sustainability of such systems.

In the past few years, plasmonic photocatalysis featuring localized surface plasmon resonance has emerged as a promising new technology that brings in some great advantages over the conventional photocatalysis previously mentioned.^7^ Particularly, the incorporation of metal nanostructures like gold could help broaden the absorption spectrum of the TiO_2_ to the visible and near-infrared and also help reduce the recombination rate by establishing a Schottky barrier at the interface.^8^ Additionally, metal nanostructures could also help enhance light absorption by scattering the incident light which increases its path length inside the hybrid film. More importantly, localized surface plasmon-assisted solar-to-fuel energy conversion is well known *via* hot-electron generation as a result of the plasmon decay process.^9,10^ Plasmonic hot electron injection is an efficient mechanism to manipulate the photoresponse of wide bandgap semiconductors and enable them to work in the visible region of the spectrum.^8,11^ The collective oscillation of charge carriers in Au nanostructures, on excitation of the plasmonic resonance, leads to the formation of highly energetic electrons that can be transferred over the Schottky barrier into the neighbouring semiconductor at appropriate interface conditions.^12–14^ These plasmonic electrons provide a new physical concept in terms of modifying the charge density of wide bandgap semiconductors and enabling them to have a photoresponse below their optical band gap, which ultimately enhances the photocatalysis process.^14^ The general principle of the photocatalysis reaction in semiconductors has been reported.^15–17^ Various plasmonic photocatalysis systems, incorporating nanostructures of different metals, have been reported such as Ag/ZnO,^18^ Au/CdSe ^19^ and Ag/TiO_2_.^20^ Gold nanostructures show a robust and efficient plasmonic response compared to other noble metals makes them highly desirable in achieving efficient photocatalysis process especially when combined with TiO_2_.

Plasmonic photocatalysis efficiency is often linked to complicated plasmonic metal structures and systems, which require state-of-the-art fabrication techniques and long processing time. For instance, Shaik *et al.* reported Au nano prisms as a plasmonic antenna with CdSe@CdS core–shell quantum dots to enhance the photocatalysis yields and kinetics.^21^ Zhao *et al.* reported an enhancement of the plasmonic photocatalytic activity by building a periodic three-dimensional nanocomposite architecture of Ag/TiO_2_ nanowires utilizing nanoimprint lithography, vertical e-beam evaporation, nano-transfer, and nano welding.^22^ Zhou *et al.* have shown a significant enhancement of the plasmonic photocatalysis of Methylene Blue dye (MB) utilizing Au/Ag NRs/TiO_2_ core–shell composite nanoparticles as a catalyst agent.^23^

Herein, a TiO_2_:Au hybrid film has been used as a catalyst to selectively degrade MB under visible light irradiation, taking advantage of the localized surface plasmonic resonance effect (LSPR) of Au nanostructures. Au nanostructured films are fabricated following a simple, fast and cost-effective technique with an annealing temperature of 300 °C in air. This work aims to provide proof of principle of a photocatalyst, which combines cost-effective fabrication and processing of plasmonic nanostructures with high photocatalysis efficiency.

## 2 Experimental methods

**Fig. 1:**
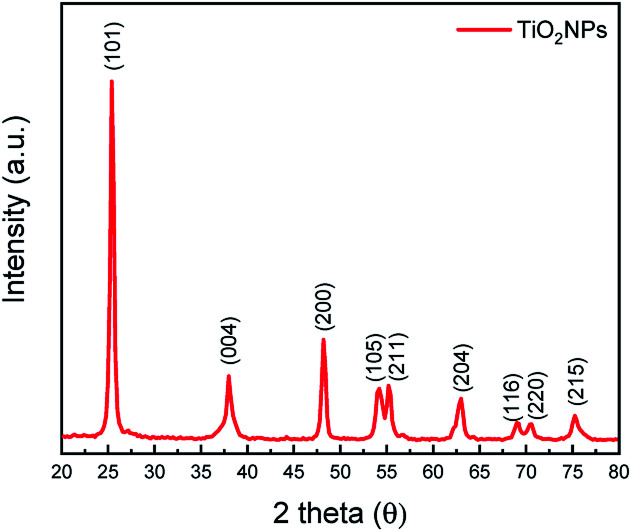
Shows XRD of TiO_2_ recorded in the powder form. The particles show a typical diffraction pattern of the anatase crystal structure.

Gold nanoislands films with a thickness of about 10 (±2 nm) nm were deposited on 2 × 2 cm^2^ glass substrates by a sputtering process. The glass substrates were cleaned thoroughly, before Au deposition, by means of an ultrasonic bath using three consecutive solutions; acetone, ethanol and distilled water and then dried with N_2_ flow. Au foil of purity 99.99% was used as a sputtering target to deposit Au nanostructured film. The sputtering process is performed using an SPC-12 compact plasma sputtering coater system with a DC current of 10 mA for the sputtering time of 20 s. The base pressure was set to 2 × 10^−4^ torr, whereas the operating pressure with inert Ar gas was 3 mTorr. The substrates were placed at a distance of 30 mm from the Au target to ensure uniform distribution of the gold atoms all over the substrate. Thermal treatments of the Au films was carried out in air using a hot plate with various temperatures and annealing times. Optical properties were measured with a UV-Vis spectrophotometer in the absorption mode at spectral range 300–900 nm. The surface morphology and roughness of the Au films were examined before and after annealing with an Atomic Force Microscope (AFM) from Angstrom working in tapping mode and images were analysed using Gwyddion (version 2.47) software. A suspension of TiO_2_ NPs (with an average diameter of 50 nm ∓ 2 nm as measured by a particle size analyser (90 Plus particle sizing software Ver.5.34) from Brookhaven Instruments) in deionised water at a concentration of 10% by weight bought from Sigma Aldrich, was deposited with thickness of 220 nm on top of the Au nanostructures annealed at 300 °C using a spin coater from HOLMARC (model: HO-TH-05) with spinning speed of 2000 rpm for 40 s. Thereafter, the hybrid film (photocatalyst) of TiO_2_:Au is baked at 60 °C for 15 m in the air to remove the solvent. The crystalline phase of the TiO_2_ NPs used in this work is studied by powder X-ray diffraction (XRD) analysis of D/max 2550 PC, Rigaku Co., Japan. Fig. 1 shows the XRD pattern of TiO_2_ utilized in this work confirming its anatase crystalline structure.

Laser light with a wavelength of 532 nm and power of 10 mW was utilized as a light source to photoexcite the hybrid photocatalytic film immersed in 2 ml of 5 ppm solution of MB in a glass container put on a stainless steel bench which act as a heat sink to help stabilize the reaction temperature. The light beam was expanded using a beam expander to cover an area of 100 mm^2^ of the plasmonic photocatalyst, resulting in irradiance of 2.3 mW cm^−2^.

Artificial sunlight (150 W Xe lamp with Air Mass 1.5 Global Filter) was used as a second light source to study the photodegradation of MB. In this case, an irradiance of 94 mW cm^−2^ was used. A UV filter has been used with the solar simulator source to block the UV light from going through and cause unwanted photoreaction process. The temperature of aqueous solution during the reaction at both light sources was in the range of 23 ∓ 2 °C. The liquid temperature is monitored using contactless IR thermometer (Fluke 62 MAX+). The photodegradation process was monitored by measuring the absorbance spectrum of MB using the UV-Vis spectrophotometer for different irradiation times (5, 10, 20, 30, 40, 50 and 60 minutes) of the laser and the artificial sunlight source. Two control experiments were also carried out only for irradiation with the laser. For the first, MB absorbance was monitored without a photocatalyst and, for the second, a TiO_2_ film on glass without Au nanoislands was used as a photocatalyst.

## 3 Results and discussion

### 3.1 Au nanoisland morphology

**Fig. 2:**
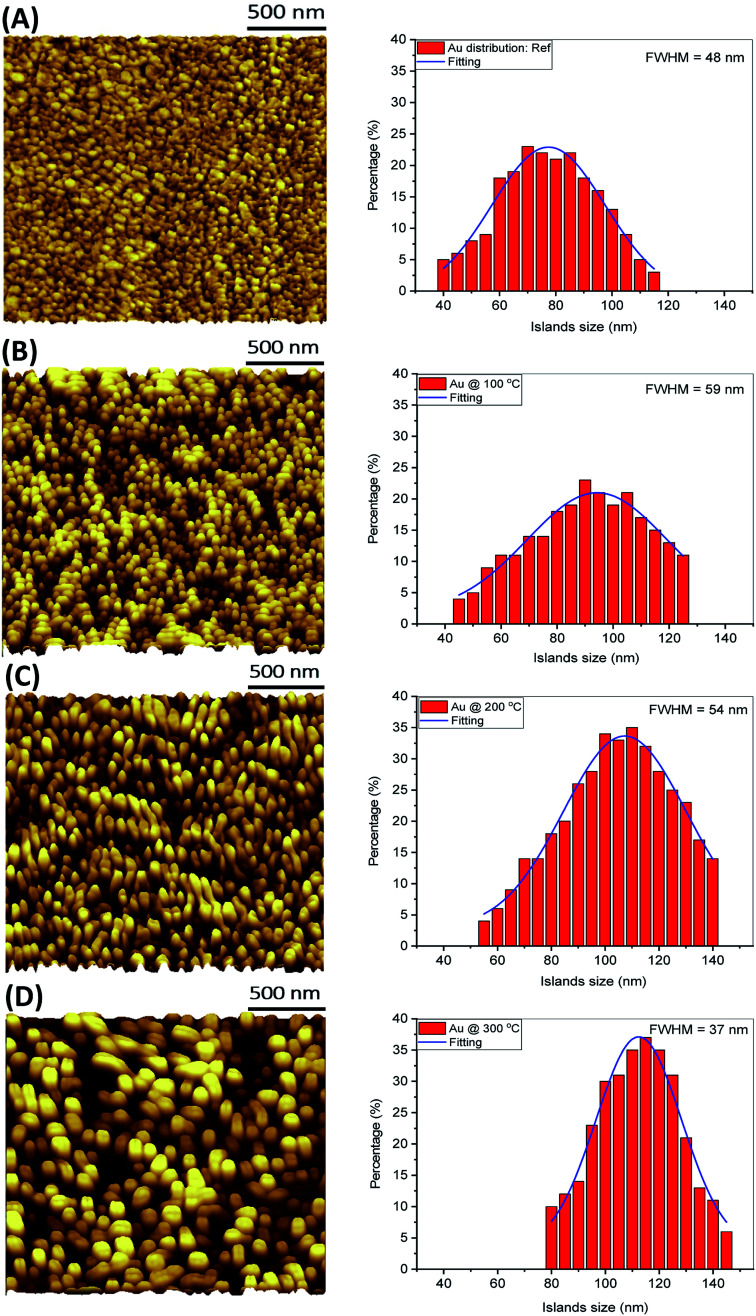
AFM images showing the surface morphology of Au nanostructured films deposited on glass substrates and treated with different annealing temperatures: (A) as-deposited, (B) 100 °C, (C) 200 °C and (D) 300 °C. The annealing is performed in air. Histograms showing the size distributions of the Au nanostructures.

It is well reported^24–26^ that heat treatment of Au thin films, deposited on poor adhesion substrates like glass, stimulates morphological changes which lead to isolated nanostructures with variable sizes and shapes. This dewetting feature could be of great benefit when trying to manipulate the surface morphology of Au thin films with minimal temperature applied. The surface profile of 10 nm (∓2 nm) thickness Au nanostructures films sputtered on glass substrates was analysed, following annealing at different temperatures as shown in Fig. 2. Fig. 2-A shows the surface morphology of Au films prior to an annealing treatment. The film shows a wide size distribution of irregular closely packed gold structures formed during the sputtering process as a semi-continuous film. The discontinuous nature of the Au film is attributed to the difference in the sticking force between Au–Au and Au–glass where the latter is found to be much weaker compared to the affinity between Au atoms.^27^ The surface morphology starts to change when the Au film is annealed on a hot plate in the air at a temperature of 100 °C for 60 min as shown in Fig. 2-B. The smaller structures start agglomerating and form ordered-like discrete structures with various diameters. The size distribution histogram shows that the average size of gold structures increases with temperature and its distribution is slightly broadened compared to the as-deposited sample.

**Fig. 3:**
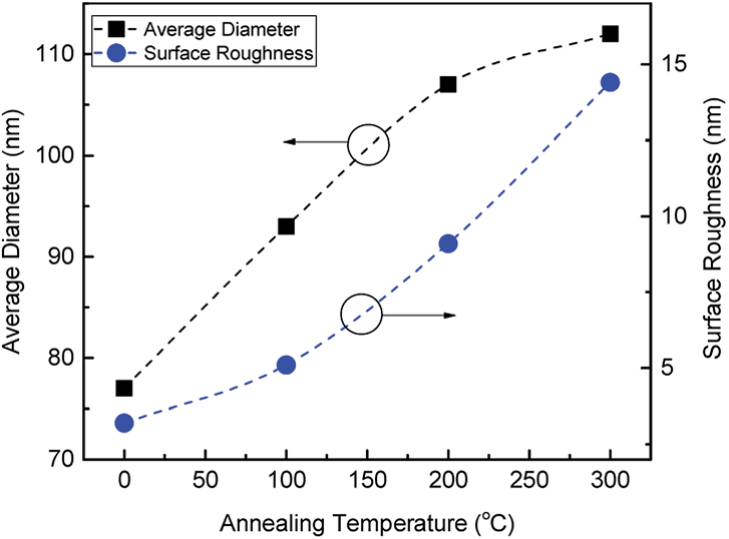
The average diameter of Au nanoislands in addition to surface roughness with different annealing temperatures.

**Fig. 4:**
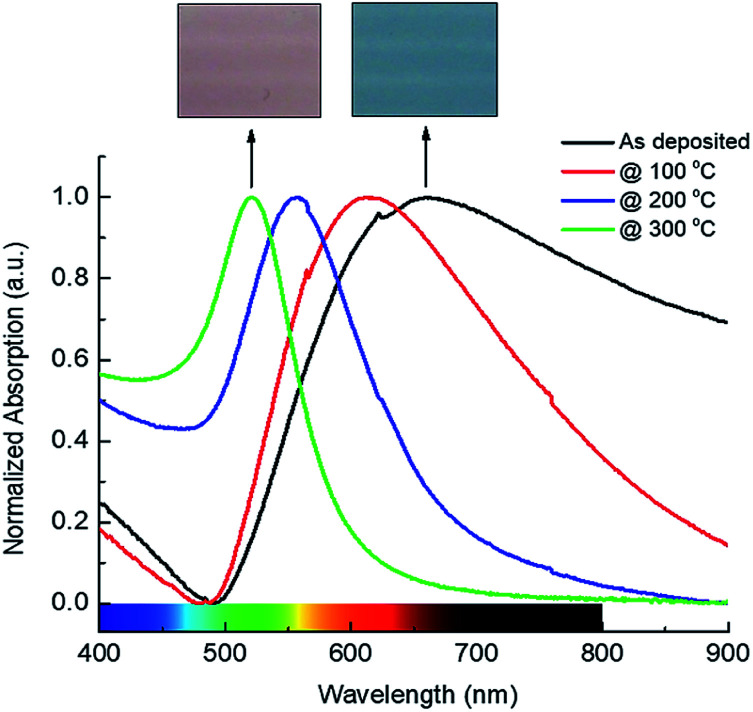
The SPR absorption of 10 nm Au thin films sputtered on glass substrates and annealed at different temperatures in air for 60 min. The inset showing optical images of Au films associated with their optical absorption spectrum.

Annealing at 200 °C leads to a drastic variation in the shape and size of the Au structures forming complex melting patterns with large diameters as shown in Fig. 2-C. The variation of the full width at half maximum (FWHM), calculated from the size distribution histograms, indicating a gold recrystallization process towards more uniform Au shapes and sizes. The surface morphology of the Au film at 300 °C is more uniform with distinct columnar structures separated by gaps. The FWHM is narrowed to 37 nm. The modifications of the Au nanoislands shape with temperature is an adaptation to reduce their surface energy. A narrowing of the size distribution is linked to the drastic change of the nanostructure colour from dark blue to reddish (can be observed by the naked eye as shown later in the inset of Fig. 4). Such a colour is attributed to a plasmonic resonance resulting from more uniform shapes of Au nanoparticles.^28^Fig. 3 summarises changes to the average diameter and surface roughness of the Au nanostructures with annealing temperature. The diameter increases from about 90 nm to about 107 nm with temperature from 100–200 °C with an increment of 14%. While further increasing the annealing temperature to 300 °C shows a slower increase of only 4%, suggesting that growth is saturating.

### 3.2 Optical properties

Electronic oscillation of the Au nanostructures gives rise to a characteristic absorption peak (mainly in the visible) attributed to the localized surface plasmonic resonance (LSPR) of surface electrons. This absorption behaviour can be characterised by a UV-Vis spectrophotometer or even can be seen by a colour change of the Au film.^29^Fig. 4 shows the normalized optical absorption spectra of the Au nanostructured thin films before and after thermal treatment. The figure clearly shows a shift in the plasmonic resonance absorption peak towards shorter wavelengths with annealing temperature.

**Fig. 5:**
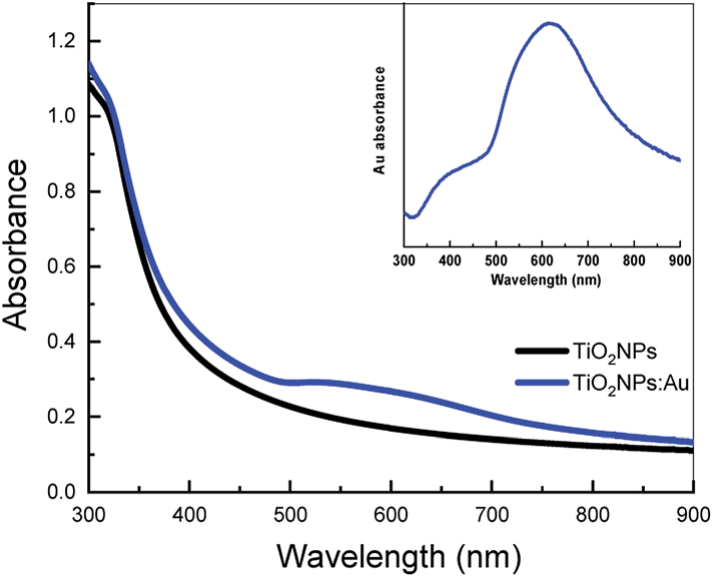
The absorption spectra of TiO_2_ and TiO_2_:Au films. The inset represents the plasmonic absorption originated from Au nanostructures incorporation calculated by subtracting the absorption of TiO_2_ from TiO_2_:Au.

It also shows a significant change in the FWHM of the absorption peak with annealing temperature. Optical absorption of the as-deposited Au film shows a broad peak with the absorption resonance at around 660 nm. Annealing at 100 °C for 1 hour in the air blueshifts the plasmonic resonance to 610 nm and narrow it to an FWHM of 219 nm. Annealing at 200 °C and 300 °C results in further narrowing of the FWHM, to 86 nm to 53 nm respectively, and a blue shift of the absorption resonance, to 555 nm and 521 nm respectively. The inset in Fig. 4 shows optical images of the Au films before (as deposited) and after annealing at 300 °C for 1 hour. The colour of the film is clearly changed from dark blue to red after annealing which supports the morphological transformation from random shaped Au islands to uniform-like Au particles shown earlier in the AFM images (Fig. 2-A and D). Moreover, our results show that annealing of 10 nm Au film at 300 °C for 60 min in the air is sufficient to obtain the almost full morphological transformation from random islands to uniform particles in much less time than previously reported.^30,31^ It is found that there is no significant difference in the resonance absorption FWHM and peak position of Au film annealed at 300 °C for 60 min and 120 min following the same annealing conditions. Fig. 5 shows the absorption spectra of two TiO_2_ thin films, one with and one without an underlying Au nanostructure layer annealed at 300 °C for 1 hour in the air prior to TiO_2_ deposition. Both spectra show a characteristic absorption peak in the UV due to the electronic interband transition in TiO_2_.

The latter also shows a broad hump in the visible due to the delocalization of electrons in the Au as a result of the plasmonic resonance absorption. The figure also shows a slight increase in the UV absorption of TiO_2_ incorporating Au nanostructures which could be attributed to the localized plasmonic field and/or to light scattering by Au inside TiO_2_ film which increases the overall absorption.^32^ TiO_2_ has a very limited photoresponse in the visible due to the presence of surface defects. However, when decorated with Au nanostructures, its photoresponse is tailored and enhanced significantly in the visible as a result of the plasmonic resonance absorption as indicated by the absorption spectrum (blue line) in Fig. 5. The inset shows the plasmonic absorption contribution calculated by subtracting the absorption of TiO_2_ from the overall absorption spectrum of TiO_2_:Au. The resonant absorption peak is broadened and red-shifted when combined with TiO_2_ film compared to its spectrum discussed earlier (the green line in Fig. 4). This is probably due to the difference in the refractive index of TiO_2_ compared to air which has a significant impact on the optical properties of Au nanostructures.

### 3.3 Plasmonic photocatalysis

**Fig. 6:**
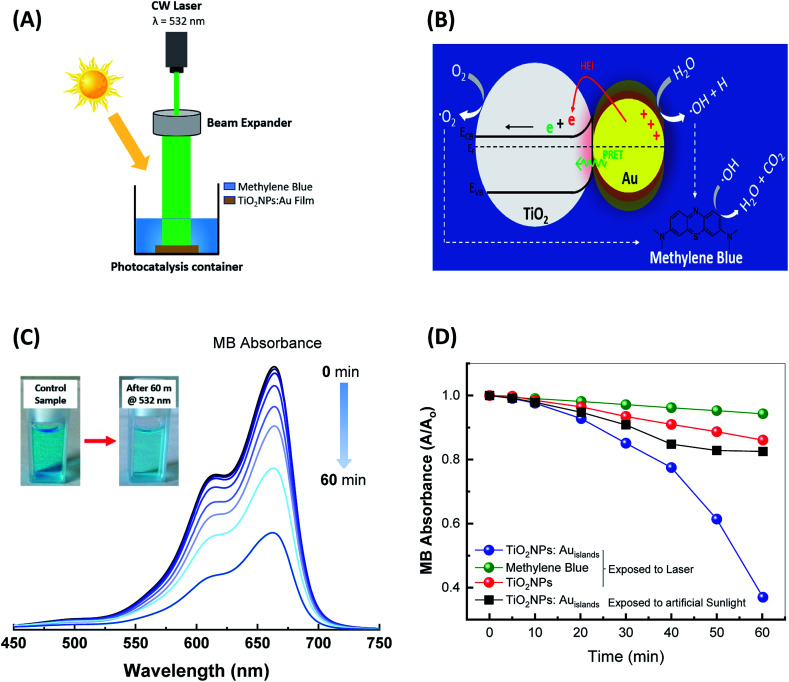
Shows (A) a schematic representation of the photocatalysis experimental set-up, (B) energy level diagram of the plasmonic hybrid structure showing possible routes for photoreaction degradation, (C) the absorption spectra of Methylene Blue dye as a function of laser irradiation time utilizing hybrid TiO_2_:Au film as a catalyst. The inset shows the MB solution before and after irradiation with the laser for 60 min. (D) The normalised absorbance of MB at 532 nm as a function of irradiation time for different configurations of photocatalyst and irradiation source.

Fig. 6-A shows a schematic representation of the photocatalysis experiment utilized in this work. Free electrons in the metal nanostructures are delocalized in response to the incident light leading to charge separation, which stimulates plasmonic absorption at the resonance wavelength. The charge separation process creates an intense internal electric field (plasmonic field) which dephases nonradiatively and creates energetic hot electrons (electrons which are not in thermal equilibrium with the metal's system). Due to the localized nature of the plasmonic effect, these electrons possess energy high enough to overcome the Schottky barrier established with the TiO_2_ and are transferred into its conduction band (CB) by hot electrons injection (HEI) leaving the Au nanoislands positively charged.^14^ Additionally, due to a possible spectral overlap between the plasmonic resonance and transitions between surface defects levels, a possible dipole coupling may be valid. This enables a second excitation source of TiO_2_, which results in generating extra electron–hole pairs due to the plasmonic resonance energy transfer process (PRET).^33^ The photodegradation process of MB could take two paths; the excess charges in the TiO_2_ conduction band resulting from plasmonic HEI could interact with oxygen in the water and create O_2_^−^ reactive species which work as a photodegradable agent to MB. The other path could results from a direct interaction of water with Au nanostructures due to the porous nature of the TiO_2_ layer. This enables Au nanostructures to balance charges by taking an electron from OH^−^ groups transfer them to hydroxyl radicals OH˙ which also help degrade MB^34^ as illustrated in the proposed charge transfer model in Fig. 6-B. The possible byproducts of MB photodegradation is already been reported in literature.^35^ The charge separation process increases the electron's lifetime and subsequently enhances photocatalysis efficiency.

Fig. 6-C shows the temporal variation of the absorption spectrum of MB examined by measuring the UV-Vis absorption of the dye periodically during irradiation in the presence of the hybrid TiO_2_:Au film as a catalyst. Additionally, a low initial concentration of MB was used and the hybrid film was kept in MB solution for 60 min prior to irradiation to reach the adsorption–desorption equilibrium following Azeez *et al.*^36^ The figure inset shows an image of the MB solution before and after irradiation with the laser for 60 min. Fig. 6-D plots the MB absorbance (*A*), at a wavelength of 664 nm, normalised with respect to the starting absorbance (*A*_o_), with irradiation time up to 60 min, when TiO_2_:Au (blue line) and TiO_2_ films (red line) are used as photocatalysts. The photodegradation profile shows a significant reduction in MB concentration, in which about 60% of the dye is degraded with time when the TiO_2_:Au hybrid photocatalyst is used as a photocatalyst compared to a minor reduction in MB concentration with the TiO_2_ film. The observed reduction in MB concentration when a TiO_2_ film is used as a catalyst is observed elsewhere^37–39^ and attributed to the surface defects which can be photoexcited with photon energy less than the semiconductor bandgap.

Most proposed plasmonic photodegradation reactions involve suspension of the plasmonic mixture in MB which is considered to be time-consuming and can only be used for one reaction at the time.^21,40^ In contrary, our method adopt a clean, fast and simple method to initiate the photoreaction by making it as a film possibly coating the wall of the container. This method does not need to separate the hybrid mixture after reaction and could potentially be reused in another reaction. Despite our simple photocatalytic configuration, our results show equivalent photodegradation performance to more sophisticated photocatalytic structures.^22,41^ The green line in Fig. 6-D shows very little degradation when the MB solution is irradiated with the laser without any photocatalyst present. This could be attributed to photobleaching.^42^ A control experiment using Au nanostructures only was not made due to the extremely fast process of plasmonic hot electrons stimulated in Au nanostructures (around 50 fs)^43^ accompanied with the small catalytic area of Au compared to the hybrid photocatalyst film.

Plasmonic excitation using a light source with a resonant wavelength is expected to enable an efficient and sustainable photocatalysis reaction compared to sunlight irradiation due to a selective initiation of the plasmonic photodegradation reaction. To better confirm this, the hybrid film was irradiated with artificial sunlight (94 mW cm^−2^) and the absorption of MB was monitored for the same irradiation period as shown in Fig. 6-D. Data show that the MB absorption is slightly reduced with sunlight irradiation and this reduction has two regions. Initially, the MB absorption reduces almost linearly with irradiation time up to 40 min when it saturates at a relatively high absorbance level. This saturation is unexpected, given the relatively high intensity of the sunlight source, which has a small but significant component of the spectrum overlapping the plasmonic resonance.

To interpret these results, we discuss the differences between the effects of the two light sources on the electronic transitions of the photocatalyst. By using the green laser, we only activate the plasmonic effect. On the other hand, only the green portion of the artificial sunlight spectrum (UV light is discarded) is resonant with the Au nanostructures. Irradiation with artificial sunlight excites a range of sub-bandgap defects states within the TiO_2_ bandgap. Some of these do not have sufficient energy to reach the conduction band and nonradiatively relax back to lower energy level, heating the lattice of the semiconductor.^44^ This photothermal effect could negatively impact the plasmonic hot electrons leading to charge collisions and energy dissipation. Other photochemical transitions may result in the generation of new traps reducing the lifetime of the surface free carriers.

## 4 Conclusions

In summary, we report a simple and cost-effective surface treatment process to tune the plasmonic absorption of Au nanostructures to stimulate visible photocatalysis reactions in TiO_2_. Our results show that thermal annealing in air at 300 °C is enough to transform surface morphology of Au nanostructured film from irregular structures to uniform islands with a defined plasmonic resonance spectrum. A hybrid photocatalyst film of TiO_2_:Au is formed by depositing a 220 nm layer of TiO_2_NPs on top of the metal nanostructures. This hybrid photocatalyst uses plasmonic hot electrons to photocatalytically photodegrade a solution of MB. A significant enhancement in photocatalytic activity of TiO_2_:Au is realized when the hybrid catalyst is irradiated at the plasmonic resonant wavelength, 532 nm, compared to a broadband artificial sunlight. We suggest that charge separation due to junction formation is vital to facilitate and sustain the photocatalysis reaction. Furthermore, we show that the photocatalysis reaction is mainly due to carrier transfer from the photocatalytic film to the MB in solution. Carrier transfer is more efficient when the photocatalyst is selectively excited at the plasmonic resonance wavelength, as competing photoreactions, which occur with broadband excitation, are avoided.

## Conflicts of interest

There are no conflicts to declare.

## Supplementary Material
